# Outcome in 12 Dogs with Chronic Radiographic Cranial Tibial Subluxation after Tibial Plateau Leveling Osteotomy (2019-2021)

**DOI:** 10.1155/2024/6681788

**Published:** 2024-05-20

**Authors:** Jacqueline M. Harrison, Peter Muir

**Affiliations:** Comparative Orthopaedic Research Laboratory, School of Veterinary Medicine, University of Wisconsin-Madison, Madison, WI 53706, USA

## Abstract

**Objective:**

The objective of this study was to examine outcomes in dogs with cruciate ligament rupture (CR) that had chronic radiographic cranial tibial subluxation at the time of osteotomy healing after tibial plateau leveling osteotomy (TPLO). *Study Design*. Retrospective case analysis of 12 dogs with prospective follow-up. Four of the 12 dogs were prospectively studied 12-24 months after surgery to assess long-term radiographic and clinical outcomes.

**Results:**

Three of the 4 dogs showed improvement in radiographic cranial tibial subluxation at long-term follow-up. In the other dog, minimally improved cranial tibial subluxation was associated with severe lameness. At long-term follow-up, gait analysis in 3 dogs with improved subluxation showed the symmetry of weight-bearing within 10% for peak vertical force, and no clinically lameness. Preoperative tibial plateau angle (TPA) and radiographic osteoarthritis in dogs with prospective follow-up and all dogs treated with TPLO surgery in the study period were not significantly different.

**Conclusion:**

Dogs with chronic radiographic cranial tibial subluxation are acceptable candidates for TPLO. Radiographic improvement in stifle reduction may take more than 10 weeks. The dog with long-term persistent subluxation also had a higher TPA over time, suggestive of ineffective surgical correction with TPLO and treatment failure.

## 1. Introduction

Cruciate ligament rupture (CR) is a common cause of lameness in dogs where progressive fiber damage in both the cranial and the caudal cruciate ligaments leads to rupture of the cranial cruciate ligament (CrCL) and occasionally both ligaments [1–3]. Surgical treatment is expensive, representing a substantial financial burden to pet owners across the world [4]. CR is a heritable polygenic disease with environmental risk factors, such as athletic activity [5–7]. Bilateral CR is common [8]. Ligament rupture occurs because of progressive fiber fraying in both cruciate ligaments over time with associated stifle osteoarthritis (OA) [1–3]. Risk of CR varies markedly amongst breeds [9]. Diagnosis of CR is based on both physical and radiographic examination findings, including pelvic limb lameness with stifle pain, stifle passive laxity indicated by a positive cranial drawer or tibial thrust test, and palpable stifle effusion. Radiographic findings include stifle effusion, osteoarthritis (OA), and cranial tibial subluxation [8, 10].

OA is a degenerative disease that develops secondary to excessive joint movement or joint inflammation [11]. A common sequela to canine CR is the development of progressive OA [12]. One aim of tibial plateau leveling osteotomy (TPLO) surgical treatment is to limit development of stifle OA by reducing dynamic cranial tibial subluxation during weight-bearing because of CrCL rupture, although progression of OA is typical [13].

CR can be managed surgically or medically [14]. TPLO is a commonly performed procedure for canine CR. However, the superiority of TPLO over other common surgical treatments, such as tibial tuberosity advancement (TTA), has not been confirmed [15]. In many patients, clinical outcomes are good to excellent, but persistence of cranial tibial subluxation after surgery remains an underinvestigated complication, and its impact on postsurgical lameness is not clear [16, 17]. The purpose of this retrospective case study was to review a consecutive series of TPLO cases with chronic postsurgical cranial tibial subluxation. We hypothesized that chronic cranial tibial subluxation at 8-10 weeks after TPLO surgery would be associated with chronic lameness within 1 to 2 years at long-term follow-up.

## 2. Materials and Methods

### 2.1. Study Population

**Figure 1:**
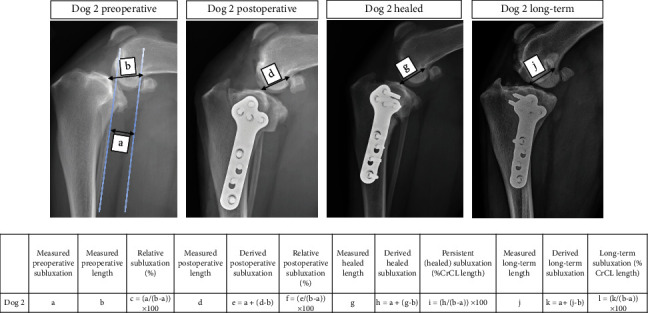
Measurement approach for cranial tibial subluxation. Length measurements (b, c, e, and h) were made as shown in included radiographs using measurement guidelines outlined by [21]. A preoperative subluxation measurement (a) was made using the method outlined by [10]. Subsequent relative subluxation measurements (values *c*, *f*, *i* and *l*) represent subluxation normalized to dog size for each visit. Normalized cranial tibial subluxation at the initial recheck (value *i*) was used as an inclusion criterion for long-term follow-up patients.

**Figure 2:**
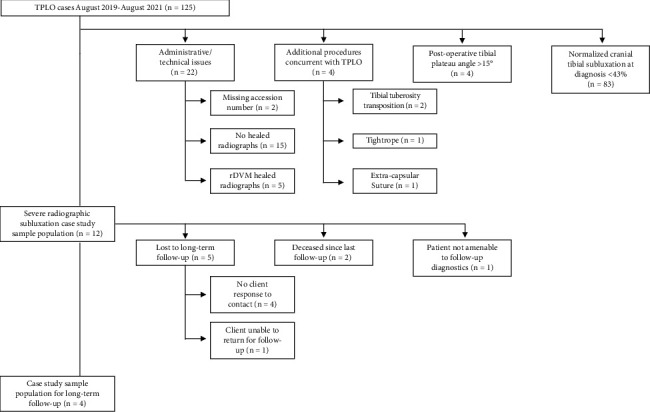
Study flow diagram outlining inclusion criteria and resulting subject number for the 12 dogs with chronic cranial tibial subluxation after tibial plateau leveling osteotomy.

Client-owned dogs that underwent TPLO stabilization at the University of Wisconsin-Madison UW Veterinary Care Hospital between August 2019 and August 2021 were evaluated. Dogs were included in this study if they had radiographically evident cranial tibial subluxation preoperatively, postoperatively after TPLO surgery, and at an 8-10-week radiographic recheck examination. Preoperative and postoperative tibial plateau angles (TPA) were measured for each dog. Dogs with severe cranial tibial subluxation at follow-up 8-10 weeks after surgery, defined by relative cranial tibial subluxation of ≥43%, calculated by measuring CrCL functional length and preoperative cranial tibial subluxation from the caudal aspect of the intercondylar fossa to the intercondylar eminence, were included (see Figure 1 for measurement details). Dogs with cranial tibial subluxation were excluded if they had a postoperative TPA of ≥15°, if they had an additional procedure performed during the TPLO surgery, if there were nondiagnostic healed recheck radiographs, or if they were lost to short-term follow-up (Figure 2). Clinical data for 12 dogs were studied in detail after review of an initial sample population of 125 dogs (Figure 2).

### 2.2. Clinical Examination

Signalment, presenting complaint, lameness severity and duration, body weight, body condition score on a 9-point scale, index pelvic limb muscle atrophy, palpable stifle joint effusion, the presence of medial buttress periarticular fibrosis, and findings from the cranial drawer and tibial thrust passive laxity tests were obtained from the medical record of each dog. Surgical findings including debridement of medial buttress, meniscectomy, and any changes in limb angulation or rotation before or after surgery were noted, including any long-term clinical and radiographic follow-up.

### 2.3. Long-Term Clinical Follow-Up

Owners of study dogs were invited to return to the UW Veterinary Care Hospital for long-term clinical and radiographic assessment of their dog at least one year after TPLO was performed. Clinical analysis included completion of an owner questionnaire investigating past and current clinical status (Supplementary File S1). An orthopaedic examination was also performed by a board-certified small animal surgeon to compare with earlier medical record findings.

### 2.4. Long-Term Follow-Up Force Platform Gait Analysis

Kinetic gait analysis was performed [18, 19]. All gait trials were performed by use of a biomechanical platform designed to measure 3D forces and impulses (OR6-6-1000 biomechanics platform with SGA6-4 signal conditioner and amplifier, Advanced Mechanical Technologies, Phoenix, Ariz.). Velocity was measured by 3 photoelectric cells mounted 1 m apart. The manufacturer of the gait analysis software reports this setup as being accurate to 0.01 m/s (Acquire v7.30, Sharon Software Inc., Dewitt, MI). The force platform system was validated for measurement of ground reaction forces (GRF) by use of weights. Photocells were validated daily for measurement of velocity by use of a pendulum. Dogs were walked and then trotted across the force platform for several trials before data collection to familiarize them with the system. Gait analysis was performed at a trot. An experienced handler guided the dog across the platform at their preferred trotting velocity. Both the handler and a trained observer with >5 years of experience evaluated each pass to confirm foot strikes and gait. The observer was positioned at the end of the gait analysis walkway with an unobscured view of the foot plate. A valid trial was defined as contact of a thoracic limb paw with the platform, followed by the ipsilateral pelvic limb paw with acceleration within 0.5 m/s^2^ at a walk. A minimum of 5 valid trials from each ipsilateral limb pair in a single session were obtained if possible. If gait analysis at the trot was not possible, standing ground reaction forces were obtained for individual limbs as appropriate.

### 2.5. Radiography

Orthogonal preoperative, postoperative, and 8-10-week recheck radiographs were evaluated. Magnification was accounted for through the use of a 10 cm measurement bar. TPA was measured using lateral stifle radiographs for each visit using a picture archiving communication system. Measurements were made using the angle between a line tangential to the central articular surface of the cranial and caudal aspects of the tibial plateau and a perpendicular line of the mechanical tibial axis. To meet the inclusion/exclusion criteria, preoperative cranial tibial subluxation for all dogs was measured as the distance between the caudal margin of the femoral condyles and the proximal caudal cortex of the fibula from the mediolateral radiograph and percentage cranial tibial subluxation was determined (Figure 1) [10]. Measurements from the preoperative, postoperative, and initial recheck radiographs after TPLO were used to identify dogs for more detailed prospective investigation. For the 12 study dogs, cranial tibial subluxation immediately after surgery and at the initial recheck was determined using changes in CrCL functional length [20, 21]. Because the study dogs differed in size, subluxation was normalized to CrCL length (Figure 1). Normalized cranial tibial subluxation at the initial recheck was used as a guideline for inclusion criteria for the prospective sample population (Figure 2), and dogs were considered to have chronic cranial tibial subluxation if normalized cranial tibial subluxation was ≥43% of CrCL length at the 8-10-week recheck visit. Stifle joint effusion (grade 0-3) and osteophytosis (grade 0-3) were subjectively graded for all dogs treated with TPLO in the study period [5, 22] using the preoperative, postoperative, and recheck radiographs. For dogs that were reexamined at long-term follow-up, measurements were repeated. Standing lateral stifle radiography was used for long-term follow-up as standing lateral stifle radiographs have been shown to yield similar CrCL length measurements compared to flexed recumbent lateral stifle radiographs, and patient sedation is not required [20, 23].

### 2.6. Data Analysis

The D'Agostino and Pearson test was used to assess the distribution of group data. Summary data were reported as mean ± standard deviation or median (range) as appropriate. An unpaired Student's *t* test or Mann–Whitney *U* test was used for group comparisons as appropriate. Results were considered significant at *P* < 0.05.

## 3. Results

### 3.1. Clinical Findings in the Study Population

There were 10 castrated males, one male, and one ovariohysterectomized female dog. Age was 5.4 ± 3.2 years and ranged from 1.3 to 10.9 years. Breeds were Pit Bull Terrier/Pit Bull Terrier mix (*n* = 4), Labrador Retriever (*n* = 2), Great Pyrenees (*n* = 2), Australian Shepherd (*n* = 2), Blue Tick Coonhound (*n* = 1), and English Bulldog (*n* = 1). The BCS was 6 (4, 7) (*n* = 11). Duration of lameness before surgery was 7 (3, 48) months. Bilateral CR was present in 8 dogs either before (*n* = 6) or after (*n* = 2) TPLO surgery of the index stifle. For dogs with contralateral CR at the time of study entry, 6 were treated surgically and 2 underwent medical management. Surgical treatment of the contralateral stifle included TPLO (*n* = 5) and lateral fabellar suture (*n* = 1). The two dogs managed medically had contralateral CR diagnosed after the study TPLO was performed. Of the 12 study dogs, there were 8 right TPLO cases and 4 left TPLO cases. All dogs included in this study had stifle arthroscopy combined with TPLO. Concurrent medial meniscectomy was performed in 10 dogs.

### 3.2. Long-Term Clinical Follow-Up

**Figure 3:**
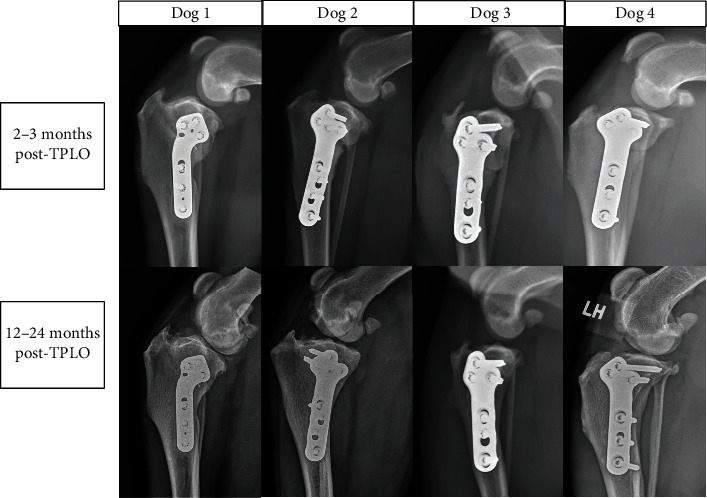
Healed and long-term follow-up lateral stifle radiographs of four dogs with chronic cranial tibial subluxation. Dog 3 had recumbent radiographs made at long-term follow-up due to difficulty attaining standing radiographs because of dog conformation. Cranial tibial subluxation was improved in dogs 2-4 at long-term follow-up. Slight worsening of the cranial tibial subluxation was found in dog 1 compared to the initial 8-10-week recheck.

Severe lameness was found in dog 1 that was considered by the owners to be like the lameness before TPLO surgery. This dog was intermittently unable to walk. Three dogs had no appreciable pelvic limb lameness at a walk or trot. All 4 dogs had palpable cranial drawer in the index stifle, but no tibial thrust on physical examination. Radiographic stifle effusion was noted in all four dogs but was improved compared with preoperative views (Table 1). Contralateral effusion was also found in one dog. Dog 1 was administered daily carprofen and gabapentin for management of postoperative index stifle pain and contralateral CR, and dog 3 was administered carprofen for pain relief as part of medical management for contralateral CR. Dog 1 also had a progressively increased TPA at healed and long-term follow-up radiographic examination compared with postoperative radiographic views: the TPA was 37° at long-term follow-up compared with 12° immediately after surgery and 24° at the recheck examination (Table 2 and Figure 3). Dog 1 developed a methicillin-resistant *Staphylococcus pseudintermedius* surgical site infection at 4 weeks after surgery that was treated with chloramphenicol. Evidence of pyoderma was present before TPLO was performed. Lameness remained static since antibiotic treatment shortly after surgery, according to owners' survey.

### 3.3. Proximal Tibial Morphology

For the preoperative radiographs, median grade of OA in the study group was 2 (2, 2) and 2 (1, 3) in the remaining dogs that were treated with TPLO during the study period (*P* = 0.06). Median grade of effusion in the study group was 3 (2, 3) and 3 (1, 3) in the remaining dogs that were treated with TPLO during the study period (*P* = 0.02). Mean TPA in the study group was 25.2 ± 6.2° and 25.1 ± 4.1° in the remaining dogs that were treated with TPLO during the study period (*P* = 0.93). For the postoperative radiographs, mean TPA in the study group was 9.5 ± 2.9° and 8.3 ± 3.5° in the remaining dogs that were treated with TPLO during the study period (*P* = 0.28).

**Figure 4:**
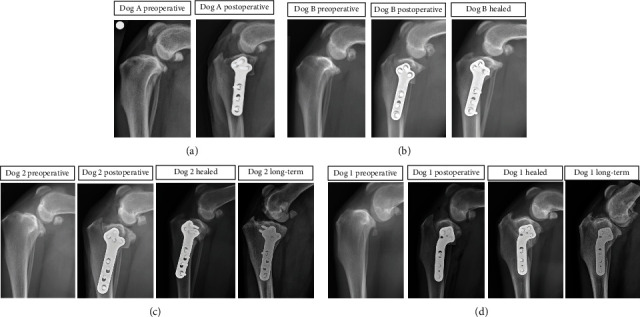
Functional outcome of dogs with cruciate ligament rupture after TPLO can be variable. (a) Appropriate postoperative reduction of cranial tibial subluxation was present on radiographs after surgery. (b) In this dog, there was cranial tibial subluxation immediately after surgery, which was resolved at the clinical recheck at 10 weeks. (c) In dog 2, reduction of chronic cranial tibial subluxation was found at long-term recheck. (d) In dog 1, cranial tibial subluxation was still present at long-term follow-up.

**Figure 5:**
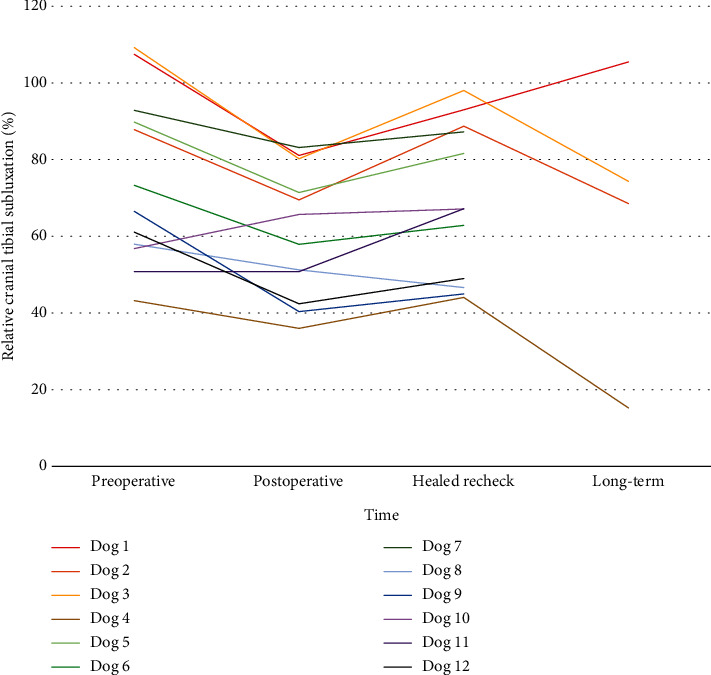
Change in relative cranial tibial subluxation (%) in 12 dogs over time. Four dogs were assessed through long-term follow-up (dogs 1-4). In all dogs other than dog 10, immediate postoperative relative % subluxation was improved. Then, relative subluxation worsened at the headed recheck evaluation. In 3 of the 4 dogs examined at long-term follow-up, subluxation further improved relative to the time of diagnosis. In dog 1, subluxation continued to worsen after the healed radiographic examination.

For the 8-10-week recheck evaluation, mean normalized cranial tibial subluxation was 67.1% (44.0, 98.0) in the study group and 21.5% (-3.0, 41.8) in the remaining dogs that were treated with TPLO during the study period (*P* < 0.001). At long-term follow-up, normalized cranial tibial subluxation had improved in 3 dogs and minimally changed in 1 dog (Table 2 and Figures 4 and 5). In the 3 dogs with improved relative cranial tibial subluxation (Figure 5), the improvement ranged from 19 to 35%. In the other dog, the subluxation was only improved by 2% (Table 2).

### 3.4. Long-Term Gait Analysis

Gait analysis results are displayed in Table 3. Gait analysis was only performed in 3 of the 4 dogs. It was not performed in dog 1 because severe lameness prevented assessment. One of the three dogs undergoing gait analysis continued to be treated with a nonsteroidal anti-inflammatory drug (carprofen ~2 mg/kg bid PRN) for suspected contralateral CR. Symmetry of weight-bearing in the index pelvic limb compared with the contralateral limb ranged from -8.3 to 1.9% for PVF and -14.8 to 2.7% for VI (Table 3).

## 4. Discussion

The present study evaluated the natural history of cranial tibial subluxation in 12 dogs with CR that were treated with TPLO. Study dogs were generally slightly overweight, adult, large breed dogs, like previous studies [9, 24, 25]. Duration of lameness was variable and was not subjectively correlated with the OA severity, in contrast to other studies of CR natural history [26].

Chronic radiographic cranial tibial subluxation in the absence of clinically detectable tibial thrust after surgical treatment is a scenario that has been recognized before [10, 27, 28]. For inclusion into this retrospective study, CrCL functional length changes were used to identify dogs with obvious cranial tibial subluxation at diagnosis and after surgery. We used 43% of CrCL functional length at the healed recheck examination as a threshold for identification with more severe subluxation before and after TPLO. In the dogs of this report with chronic subluxation, TPA before and after TPLO was not significantly different from the group of TPLO dogs without chronic subluxation, suggesting that this problem is likely explained by periarticular fibrosis, muscle tone, and functional loading rather than a high TPA value or insufficient correction of the TPA by TPLO. Four of the 12 dogs were available to us for long-term evaluation: the results and discussion primarily evaluate these four individuals, but measurement trends of all 12 dogs examined are included in Table 2 and Figure 5. At long-term recheck, cranial drawer laxity was still palpable in all 4 dogs, but tibial thrust was absent. Chronic subluxation greater than 43% of CrCL functional length remained in 3 of the 4 dogs although this was not necessarily associated with clinical lameness. Two of these dogs were doing well clinically with improved reduction of the stifle and no concerns from the owners at home despite persistent cranial tibial subluxation that was improved from the 8-10-week postoperative recheck visit. In another dog doing well clinically, improvement in stifle reduction was even more obvious compared with the initial recheck examination. These novel observations suggest that periarticular fibrosis had gradually remodeled over time in response to the alteration in stifle biomechanics induced by the TPLO procedure.

In CR dogs, periarticular fibrosis develops because of dynamic laxity of the stifle resulting in medial buttress fibrosis, which can be severe in chronically affected dogs. Clinically, detection of cranial drawer and tibial thrust laxity can be more difficult in these patients [10]. Such changes are associated with progressive stifle OA [22]. In the present study, severity of stifle effusion was increased in the study dogs with persistent subluxation, compared with the remaining dogs that were treated with TPLO during the study period.

Gait analysis of the 3 dogs doing well at long-term follow-up confirmed reasonable symmetry of weight-bearing in the pelvic limbs consistent with the owner observations which are based more on behavior than physiologic gait changes [29]. Gait analysis was performed at a trot rather than a walk, as trotting has been shown to yield more accurate force plate results than walking in dogs with low-grade pelvic limb lamenesses [30]. Bilateral CR is common in dogs [5] and was present in three of the four dogs reexamined at long-term follow-up. Dog 3 had contralateral CR managed medically, but little evidence of lameness.

At long-term follow-up, we found that one dog with a chronic history of lameness before TPLO developed progressive worsening of normalized cranial tibial subluxation over time and was presented with severe lameness. A postoperative surgical site infection at 4 weeks improved with antibiotic treatment but lameness remained static over time after treatment of the infection. The long-term efficacy of TPLO surgery in dogs is not correlated with development of surgical site infection, if the infection is treated appropriately [31]. Past research has shown that dogs with a postoperative TPA of >14° have reasonable weight-bearing [32]: in this dog, the TPA was 24° at the initial recheck and 37° at long-term follow-up. Progressive worsening of the TPA may be secondary to construct instability and rock-back [33, 34] or secondary to remodeling of the subchondral plate because of chronic severe cranial tibial subluxation. The rock-back phenomenon is more common in dogs with high TPA values before surgery [35].

We found that 11 of the 12 dogs examined showed initial radiographic improvement in cranial tibial subluxation, except for dog 10 which had higher subluxation immediately after surgery with mild worsening at 8-10 weeks after surgery. Dog 10 had a postoperative TPA of 3° and no noted tibial torsion or rotation in surgical reports. This trend may be secondary to artefactual preoperative reduction during radiography. A controlled prospective clinical trial is recommended to further analyze how TPLO influences cranial tibial subluxation over time in a larger population of dogs. Assessment of collateral laxity before and after TPLO would also be an important consideration.

Limitations of this case report study were associated with the small sample size. There are alternative approaches to estimation of cranial tibial subluxation that could have been used such as calculation of a ratio in cranial cruciate functional length between time points. The method we describe was the most reliable in our hands to provide these estimations. Use of different inclusion criterion for normalized cranial tibial subluxation could have influenced the study group which had more severe cranial tibial subluxation that persisted after surgery. Clinical evaluations were performed by different clinicians because of the retrospective nature of the study. However, orthopaedic examination findings are reasonably consistent amongst evaluators [36]. Several dogs developed bilateral CR over time, as it is common with this disease [5, 8] and this complicated patient assessment. Long-term medical treatment may also have influenced clinical signs of lameness but is unlikely to have influenced severity of cranial tibial subluxation. This study used standing stifle radiographs without sedation at long-term follow-up to maximize owner participation. Functional CrCL length measurements are not significantly influenced by patient positioning [20], but measurements performed in this study did not account for potential changes in stifle angulation between radiographs. The surgical site infection that developed in dog 1 after surgery could have influenced long-term functional outcome. This dog may also have developed postliminal meniscal damage. A prospective clinical study is recommended to further study assumptions made between radiographic positioning, radiographic measurements, and outliers in the patient population. Ideally, a prospective study would only recruit large breed dogs comfortable with gait analysis to eliminate the need for comparison between static standing forces and dynamic trotting forces using standing radiography for all examinations.

In conclusion, severe cranial tibial subluxation and periarticular fibrosis in dogs with chronic CR could be considered a contraindication for surgical treatment or at least that this type of patient is not an ideal candidate for TPLO. Our findings suggest that TPLO treatment in such dogs can induce gradual improvement in stifle joint alignment that develops slowly over weeks to months, likely because of gradual remodeling of periarticular soft tissues, although some subluxation may persist. Three of the 4 dogs at long-term follow-up in this study had reasonable limb function, suggesting that TPLO could rationally be used in this scenario to salvage patients with chronic CR and periarticular fibrosis, contrary to our original hypothesis. More investigation of this phenomenon is needed using a larger patient sample size based on the findings from this case study.

## Figures and Tables

**Table 1:** Objective and subjective data for twelve dogs with chronic cranial tibial subluxation after TPLO.

Dog	Signalment	Procedures	History (preoperative)	Contralateral CrCL rupture	Buttress/drawer/thrust/lameness grade (preoperative)	OA grade (osteophytosis/effusion) preoperative	Persistent lameness/complications (postoperative)	Lameness grade and long-term exam	OA grade (osteophytosis/effusion) long term
1	3 yr. MC Coonhound	R TPLO and medial meniscectomy	RPL lame > 1 yr.	Yes: L TPLO one year prior	+/+/+ grade 3/5 RPL lame	2/3	MRSP implant infection	RPL and LPL lameness, mild drawer bilateral, grade 4/5	3/2
2	2 yr. MC Great Pyrenees	R TPLO and medial meniscectomy	RPL lame × 5 − 6 mo.	No	+/+/+ grade 2/5 RPL lame	2/3	None	No lameness, mild drawer bilateral	2/1
3	2 yr. MC English Bulldog	R TPLO and medial meniscectomy	RPL lame × 3 mo.	Yes: current medical management	+/+/+ grade 2/5 RPL lame	2/2	None	No lameness, mild drawer bilateral	2/1
4	6.5 yr. MC Pitbull Terrier	L TPLO and medial meniscectomy	LPL lame × 10 mo. (2 mo. NWB)	Yes: TPLO 2 years earlier	+/+/+ grade 3/5 LPL lame	2/3	None	No lameness, mild drawer bilateral	2/1
5	9 yr. MC Labrador Retriever	R TPLO and medial meniscectomy	RPL lame × 3‐4 yr. (progressive past 1 yr.)	No	+/+/+ grade 3/5 RPL lame	2/3	None	Not performed	N/A
6	2 yr. MC Labrador Retriever X	R TPLO and medial meniscectomy	RPL lame × 1 yr.	Yes: L lateral suture > 2 years prior	+/+/+ grade 3/5 RPL lame	2/3	MRSP surgical site infection	Not performed	N/A
7	11 yr. MC Pitbull Terrier	R TPLO and medial meniscectomy	RPL lame × 3 mo.	Yes: L TPLO 6 months after R TPLO	+/+/+ grade 3/5 RPL lame	2/3	Acute lameness 2 weeks after surgery, improved with antibiotics	Not performed	N/A
8	5.5 yr. MC Pitbull Terrier	R TPLO	RPL lame × 3 mo.	Yes: L TPLO 6 months after R TPLO	+/+/+ grade 2/5 RPL lame	2/3	None	Not performed	N/A
9	6 yr. MC Great Pyrenees X	L TPLO and medial meniscectomy	LPL lame × 3 mo.	Yes: R TPLO 2 years earlier	+/+/+ grade 3/5 LPL lame	2/3	None	Not performed	N/A
10	5 yr. MC Labrador Retriever X	L TPLO	LPL lame × 1.5 yr.	Yes: current medical management	+/+/+ grade 3/5 LPL lame	2/3	None	Not performed	N/A
11	9.5 yr. FS Australian Cattle Dog	L TPLO and medial meniscectomy	LPL lame × 3 mo.	No	+/+/+ grade 3/5 LPL lame	2/3	None	Not performed	N/A
12	11 yr. MC Pitbull Terrier	R TPLO and medial meniscectomy	RPL lame × 8 mo.	Yes: L TPLO 1 year earlier	+/+/+ grade 3/5 RPL lame	2/3	None	Not performed	N/A

Note: CrCL: cranial cruciate ligament; R: right; L: left; OA; osteoarthritis; yr.: year; MC: castrated male; TPLO: tibial plateau leveling osteotomy; RPL: right pelvic limb lameness; LPL: left pelvic limb lameness; MRSP: methicillin-resistant *Staphylococcus pseudintermedius*; mo.: month old; N/A: not applicable; NWB: non-weight-bearing. Lameness was graded on a five-point scale. OA and effusion were graded on a three-point scale. Data were collected from medical records from the preoperative and postoperative period as well as surgery reports.

**Table 2:** Radiographic findings in 12 dogs with persistent cranial tibial subluxation after TPLO.

Dog	Before surgery				After surgery				Healed recheck				Long-term recheck			
	(a) Cranial tibial subluxation (mm)	(b) CrCL length (mm)	(c) Relative subluxation (%)	TPA (°)	(d) CrCL length (mm)	(e) Cranial tibial subluxation (mm)	(f) Relative subluxation (%)	TPA (°)	(g) CrCL length (mm)	(h) Cranial tibial subluxation (mm)	(i) Relative subluxation (%)	TPA (°)	(j) CrCL length (mm)	(k) Cranial tibial subluxation (mm)	(i) Relative subluxation (%)	TPA (°)
1	21.6	41.7	107	27	36.4	16.3	81	12	38.8	18.7	93	24	41.3	21.2	105	37
2	18.7	40	88	35	36.1	14.8	69	14	40.2	18.9	89	20	35.9	14.6	69	20
3	16.6	31.8	109	33	27.4	12.2	80	10	30.1	14.9	98	11	26.5	11.3	74	10
4	10.2	33.8	43	25	32.1	8.5	36	8	34	10.4	44	12	27.2	3.6	15	11
5	17.6	37.2	90	31	33.6	14	71	9	35.6	16	82	6				
6	16.2	38.3	73	16	34.9	12.8	58	15	36	13.9	63	16				
7	18.2	37.8	93	25	35.9	16.3	83	10	36.7	17.1	87	20				
8	16.4	44.7	58	23	42.8	14.5	51	15	41.5	13.2	47	17				
9	14.5	36.3	67	14	30.6	8.8	40	9	31.6	9.8	45	11				
10	12.1	33.4	57	21	35.3	14	66	3	35.6	14.3	67	4				
11	9.6	28.5	51	26	28.5	9.6	51	11	31.6	12.7	67	14				
12	15.7	41.4	61	26	36.6	10.9	42	10	38.3	12.6	49	20				

Note. CrCL: cranial cruciate ligament; TPA: tibial plateau angle. Cranial tibial subluxation was measured using the method of [10]. CrCL length was measured using the method of [20]. Relative subluxation was calculated as a percentage of CrCL length. Dogs 5-12 were not available for long-term follow-up.

**Table 3:** Peak vertical force and impulse gait measurements at long-term follow-up in three dogs with cranial tibial subluxation after TPLO.

Dog	PVF (N/kg) right pelvic limb	PVF (N/kg) left pelvic limb	PVF symmetry index (%)	VI (N-sec/kg) right pelvic limb	VI (N-sec/kg) left pelvic limb	VI symmetry index
2	5.22^∗^	5.67	-8.3	2.12^∗^	2.1	1.0
3	1.59	1.54^∗^	-3.2	3.2	2.76^∗^	-14.8
4	6.09^∗^	5.98	1.9	1.86^∗^	1.81	2.7

Note. PVF: peak vertical force. At long-term follow-up, dog 1 was severely lame and nonambulatory without sling support, so collection of ground reaction force data was not possible. In dog 3, standing data were obtained because the stride length and dog size were too small to obtain gait data at the trot. ^∗^Index stifle treated with tibial plateau leveling osteotomy (TPLO). Symmetry index was calculated using a standard method [37].

## Data Availability

The clinical data used to support the findings of this study are included within the article.

## Supplementary Materials

Supplementary Materials Supplementary File S1: client questionnaire for long-term follow-up of dogs with chronic CR.
